# Accurate SPARQL generation via in-context learning and schema-based query construction

**DOI:** 10.1093/bioinformatics/btag174

**Published:** 2026-04-08

**Authors:** Hikaru Nagazumi, Yuki Moriya, Shuichi Kawashima, Toshiaki Katayama, Kana Shimizu

**Affiliations:** Faculty of Science and Engineering, Waseda University, Shinjuku-ku, Tokyo 169-8555, Japan; Database Center for Life Science, Kashiwa, Chiba 277-0871, Japan; Database Center for Life Science, Kashiwa, Chiba 277-0871, Japan; Database Center for Life Science, Kashiwa, Chiba 277-0871, Japan; Faculty of Science and Engineering, Waseda University, Shinjuku-ku, Tokyo 169-8555, Japan

## Abstract

**Motivation:**

Integrated analysis across biological databases is becoming increasingly important in life science research, leading many public databases to adopt Semantic Web technologies, also known as knowledge graphs. However, biological data possesses inherently complex and diverse structures, which makes the resulting Resource Description Framework (RDF) schemas intricate and difficult for non-expert users to master, preventing them from translating natural language questions into correct SPARQL queries. Although recent large language model (LLM)-based approaches show potential for automatic SPARQL query generation, they often suffer from structural hallucinations and require large-scale training data to capture schema-specific structures. In this study, we propose a novel framework that avoids hallucinations and requires no training data by combining LLM-based word extraction with a schema-based SPARQL query builder.

**Results:**

The LLM extracts variables and parameters from the user’s question based on a predefined schema, and the query builder generates a syntactically correct SPARQL query accordingly. By providing a predefined schema in prompts, our method eliminates the need for training data. Experimental results on UniProt, Rhea, and Bgee demonstrate that our method outperforms baseline LLM-based methods using fine-tuning and prompt-tuning in terms of the similarity between search results obtained from generated and expert-written queries. Furthermore, we developed a proof-of-concept chatbot system that enables users to query RDF databases via natural language input, demonstrating the practical utility of our approach in improving access to biological data resources.

**Availability and implementation:**

Experimental environment: https://github.com/scott2121/sparql_query_generator (DOI: https://doi.org/10.5281/zenodo.18539213). Chatbot: https://github.com/scott2121/sparql_query_chatbot (DOI: https://doi.org/10.5281/zenodo.18539225).

## Introduction

Modern life sciences research requires integrated analyses across diverse datasets to understand complex biological phenomena, particularly in drug discovery where a comprehensive knowledge graph in biomedicine is essential for identifying therapeutic targets and developing new treatments. To facilitate this, public databases, serving as essential research infrastructure, should ideally be unified and integrated regardless of their repository locations, so that scientists can fully leverage available data resources. Semantic Web (SW) technologies (Antoniou and Van Harmelen 2004), also known as knowledge graphs (Cimiano and Paulheim 2017), provide standardized data formats and web-based access protocols established by the W3C, enabling unified data representation and interoperability. These technologies have been widely adopted by major life science databases including UniProt (Jain et al. 2009) and wwPDB (Kinjo et al. 2012), allowing seamless cross-database searches and integration. In the SW, data are described using the Resource Description Framework (RDF), and RDF datasets are queried with the SPARQL query language. RDF’s schema-flexible nature enables integration of heterogeneous biological datasets, potentially addressing a wide range of research questions through cross-database analysis. However, this flexibility entails significant challenges, as researchers must understand and navigate the diverse and complex schemas of biological data. Moreover, significant effort is required to comprehend the diverse schemas of an increasing number of datasets. To utilize abundant resources while avoiding the cost of query generation, a series of studies has investigated automatic SPARQL query generation from natural language questions. Among them, BioSODA (Sima et al. 2022) proposed automatic query generation based on a graph-based approach. Also, recent advancements in large language models (LLMs) encourage approaches such as by fine-tuning (Rangel Reyes et al. 2024) and by RAG (Emonet et al. 2024) to generate SPARQL query by using pairs of natural language questions and corresponding SPARQL queries. Although both graph-based and LLM-based methods have the advantage of reducing the manual effort required for implementing rules, the former may overlook the intentions of the schema designer, while the latter requires a vast amount of data to implicitly capture the underlying schema embedded in the datasets. Additionally, the LLM-based approach is prone to hallucinations, even when generating grammatically correct queries.

To precisely capture the underlying schema while avoiding the need for extensive training data preparation, we propose a novel approach that combines LLM-based word extraction with a schema-based SPARQL query builder. The approach separates the query synthesis into two tasks: (i) word selection that is consistent with the DB schema and the user’s question, and (ii) query generation that strictly follows the language syntax. As a result, the role of the LLM is limited to extracting necessary information from the question text, taking the user’s intent into account. We use RDF-config (https://github.com/dbcls/rdf-config; accessed in June 2025) as a schema-based query builder that generates SPARQL queries from human-readable variable names and their parameters. In our method, the LLM selects question-related variables from the schema defined in RDF-config and extracts relevant parameters from the question text. Then, RDF-config generates a syntactically correct SPARQL query. It should be noted that the LLM in our method only needs to learn RDF-config’s schema, whereas LLMs in previous studies are expected to understand both complex database schemas and SPARQL syntax only from examples.

We implemented our method and compared it with two baseline methods, in which an LLM generates SPARQL queries either through fine-tuning on a question-query dataset or via prompt tuning using the database schema. The experiments conducted on question-query datasets created for UniProt (Jain et al. 2009), Rhea (Bansal et al. 2022), and Bgee (Bastian et al. 2021) demonstrated that the proposed method outperformed all baseline methods in terms of accuracy. The accuracy was measured by the similarity (Jaccard coefficient) between the search results obtained from the generated SPARQL queries and those from expert-written SPARQL queries. We also developed a proof-of-concept chatbot system that enables users to search an RDF database through natural language question answering.

## Method

**Figure 1:**
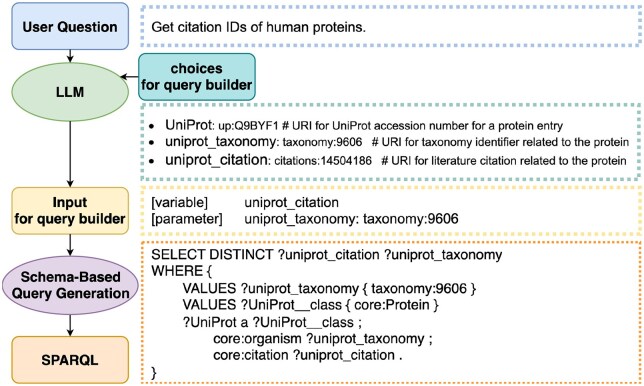
The overview of our approach. The LLM generates variables and parameters from the user’s question and database choices, where parameters are variable-value pairs. RDF-config then generates a SPARQL query from these variables and parameters. The output of LLM is converted to SPARQL through schema-based query generation.

We propose a novel approach that combines word extraction using an LLM with SPARQL query generation using a schema-based query builder. Figure 1 illustrates an overview of our method. Given a natural language question, the LLM generates relevant *variables* and *parameters* (variable-value pairs) that serve as input to the query builder RDF-config. RDF-config operates on a predefined database schema represented as a set of triples, where the subject and object are treated as variables. These variables are used in the resulting SPARQL query. The LLM maps the question to schema-defined variables and extracts binding values (such as entities or literals), forming parameters (variable-value pairs), while resolving ambiguities and handling informal input based on context. The resulting variables and parameters are passed to RDF-config, which generates syntactically valid and structurally consistent SPARQL queries. This hybrid approach does not require the LLM to have knowledge of SPARQL syntax and facilitates the isolation of LLM-induced errors, thereby simplifying query correction.

### RDF-config

RDF-config is a schema-based query builder developed to address the complexity of determining property paths to retrieve specific information from complex graph structures. RDF-config simplifies this process by using manually curated schemas, from which schema diagrams and SPARQL queries can be automatically generated. In this context, an RDF model refers to the inherent data structure within RDF data as designed by its creators. An RDF-config schema describes this RDF model in a simplified tree structure, interpreted and annotated by domain experts to make the complex graph structure more accessible. Life science databases represented in RDF contain the same information as original database entries, where key entities (e.g. proteins in UniProt, genes in Ensembl, chemical compounds in PubChem) serve as subjects with their diverse biological or chemical attributes represented as objects connected through appropriate predicates. In RDF-config, each dataset is associated with a schema file that defines an RDF model focusing on key property paths from the original graph structure. While the complete RDF data forms complex graph structures with nested predicates and multi-step relationships, these schema files accurately reflect this complexity by representing the hierarchical property paths as tree structures that preserve the original graph relationships.

**Figure 2:**
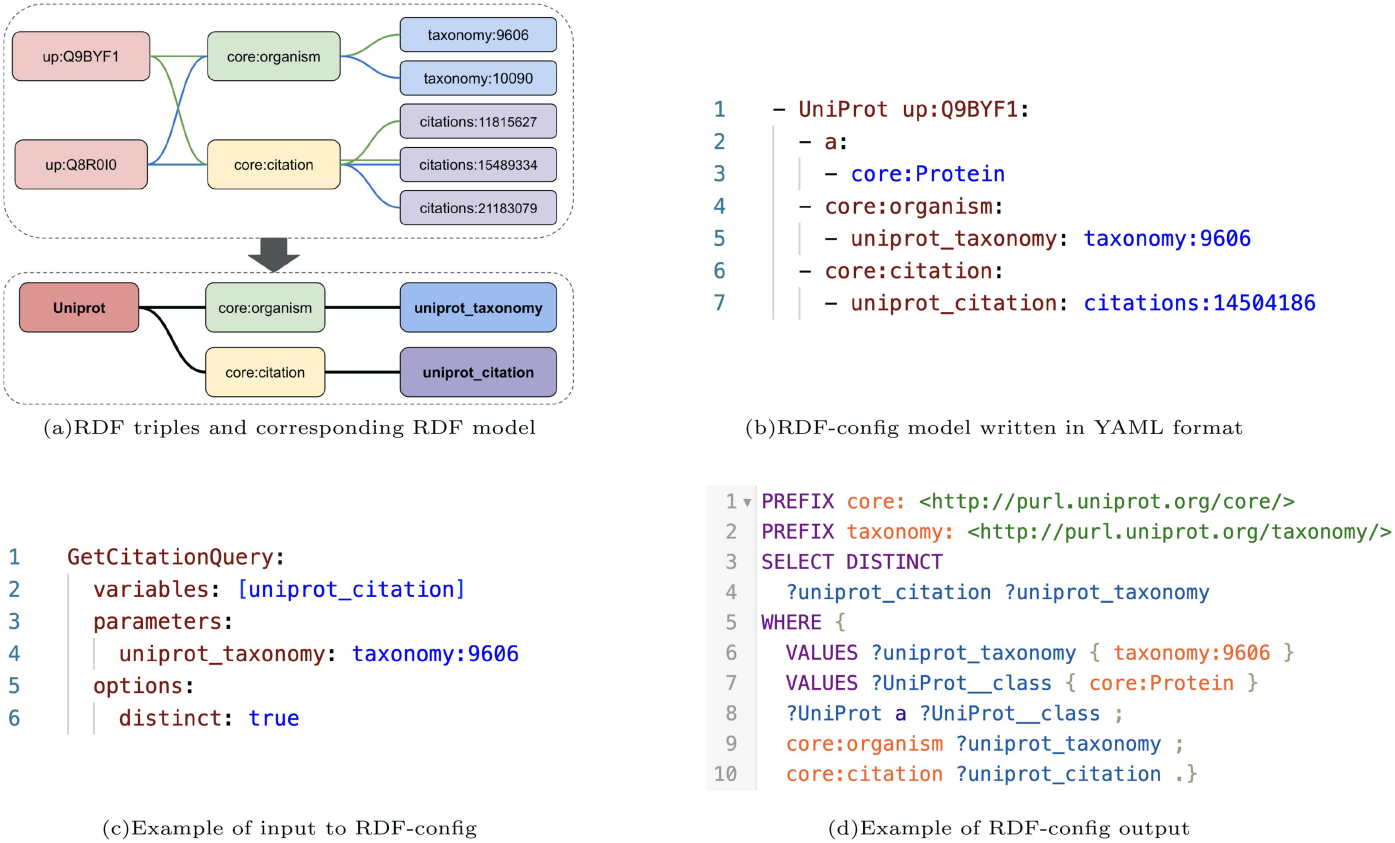
Transformation of graph patterns into RDF-config model and SPARQL query generation. (a) RDF triples and the corresponding RDF model showing two important triple patterns: (1) protein ID → organism → taxonomy ID and (2) protein ID → citation → citation ID. Nodes with a common role are merged into a single node in the schema and treated as a variable. (b) The RDF-config model written in YAML format, where UniProt (representing protein ID) serves as the root node of the tree structure, with uniprot_taxonomy (taxonomy ID) and uniprot_citation (citation ID) as child nodes. Example values are provided next to each variable name (e.g. up: Q9BYF1 for UniProt) to help understand the meaning of the variable. (c) Example of input to RDF-config for retrieving citation IDs from human (taxonomy: 9606) proteins, where uniprot_citation is the required variable and uniprot_taxonomy with taxonomy: 9606 forms the required parameter (variable-value pair). (d) Example output from RDF-config showing the SPARQL query generated from the input in (c) to obtain citation IDs from human proteins.

Consider a typical biological database, which consists of a collection of database entries. When represented in RDF triples, since each entry generally follows the same RDF data structure, a single schema can represent the common pattern. Through manual curation, human-readable variable names are assigned to subjects and objects in this schema that are not present in the original RDF model. For example, in UniProt, the subject might be assigned the variable name “UniProt” while objects receive descriptive names like “uniprot_taxonomy” for taxonomic information. These variable names are unique within each RDF schema and designed for human comprehension. Sample values corresponding to subjects and objects are also included in the schema to help users intuitively understand variable usage. As illustrated in Fig. 2(a), each entry follows the same RDF data structure. The RDF-config schema represents the common subgraph patterns corresponding to database entries found within the graph as an RDF model. Figure 2(b) shows the corresponding RDF-config schema written in YAML format for the data structure illustrated in Fig. 2(a). (see Section 2, available as supplementary data at *Bioinformatics* online for more details on constructing RDF-config schema.)

### SPARQL query generation with RDF-config

Given a schema file created by a database expert, RDF-config allows users to generate appropriate SPARQL queries by specifying variables and parameters, where variables correspond to subjects or objects, and parameters correspond to variable-value pairs. Variable names defined in the schema are carefully chosen to reflect their meaning, enabling users to easily select the necessary information from the diverse data contained in the database without knowing the underlying RDF structure details. Figure 2(c) shows an example of a user query that specifies variables and parameters. The variables indicate items to retrieve in the SELECT clause, while parameters are used in the VALUES statement of the WHERE clause. By traversing the tree from the root to the selected variables, RDF-config generates necessary property paths to be included in the resulting SPARQL query. Figure 2(d) shows the corresponding output from RDF-config.

Many life science RDF datasets are stored in RDF Portal (https://rdfportal.org), and their schemas are available in the RDF-config GitHub repository (https://github.com/dbcls/rdf-config/tree/master/config; accessed in June 2025). As of November 2025, the repository contains 80 database models, so users can generate SPARQL queries without writing schemas for these databases. We encourage the community to contribute new schemas, so that RDF-config can support a wider range of existing databases. Also, an example use of RDF-config can be seen in the PubChem RDF schema (https://pubchem.ncbi.nlm.nih.gov/docs/rdf-schema; accessed in June 2025). The queries generated by RDF-config are limited to simple searches and do not support aggregation functions such as COUNT or GROUP BY. However, we consider that these limitations can be addressed by modifying the queries using an LLM in the post-processing stage. In the Application section, we demonstrated a case where the initially generated SELECT statement was later modified into a COUNT statement.

### Generating input to RDF-config using an LLM

**Figure 3:**
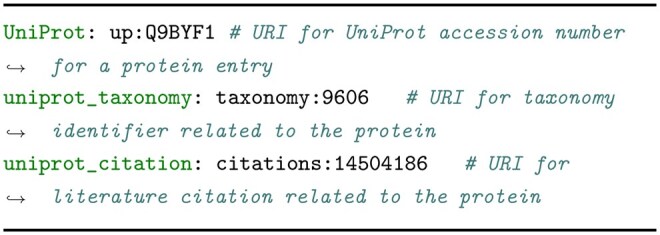
Variables and their explanations used in Fig. 2(b).

**Figure 4:**
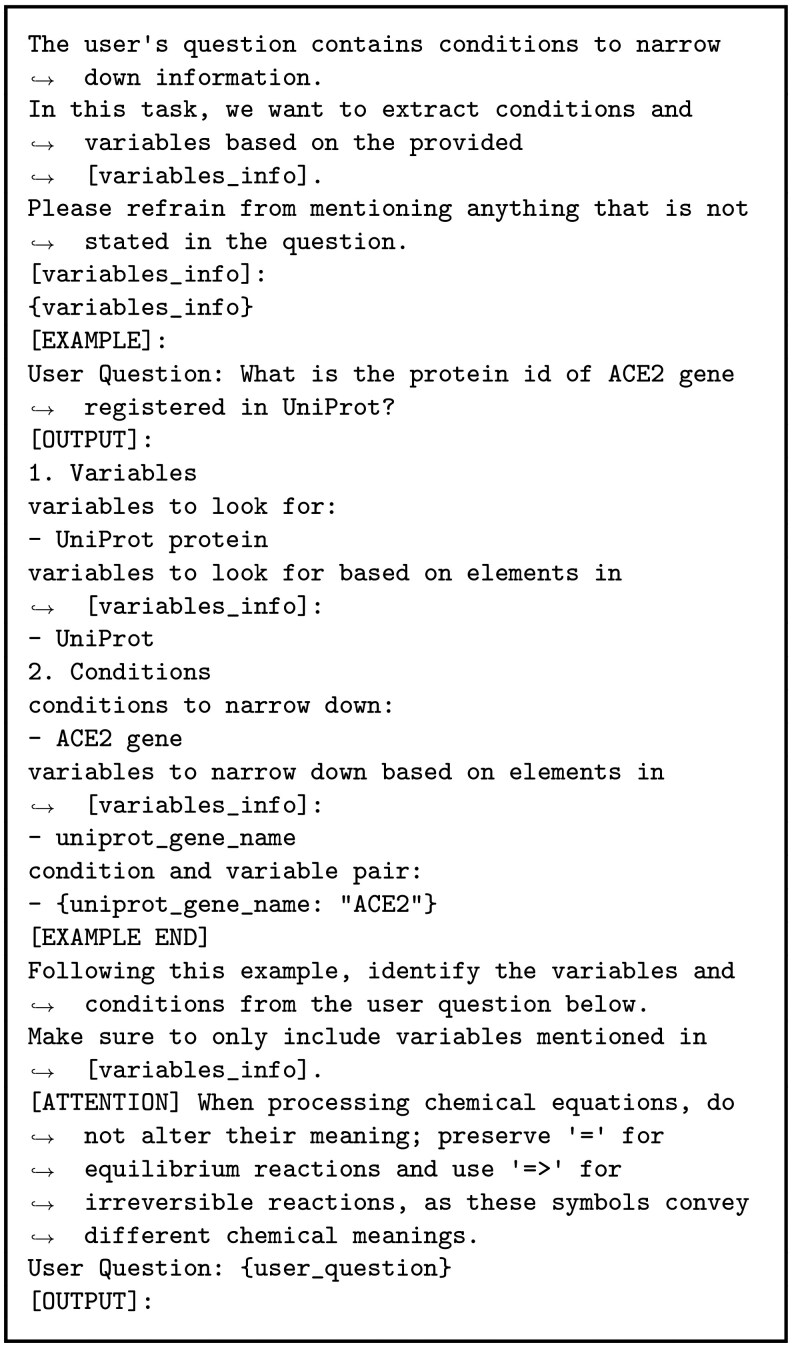
Prompt for extracting information from user questions.

The input to RDF-config consists of variables and parameters. To generate appropriate input for a given natural language question, the LLM needs to understand the meanings of the variables defined in the RDF-config schema. To simplify this task, we provided a list of variables along with auxiliary information, rather than the whole schema file. More precisely, in our method, the LLM receives two inputs in the prompt shown in Fig. 4: a natural language question (inserted at {user_question}) such as “What is the protein ID of ACE2 gene registered in UniProt?”, and a set of variable descriptions (inserted at {variables_info}) as shown in Fig. 3. Each variable description consists of example values and a brief explanation of the variable, where we manually add explanations for the use of variables. In fact, the quality of the generation is affected by how schema information is presented, and we found that this approach achieved higher accuracy than using the full schema. (See the Results section for more details.) Notably, since RDF-config maintains the full schema and resolves structural model details, supplying only a list of variables is sufficient for our method. Moreover, we retained only semantically meaningful variables, excluding variables used solely as intermediate connector nodes, since these are also resolved internally.

When processing these inputs, the LLM employs a Chain of Thought prompting method (Wei et al. 2022) that breaks down the task into four distinct steps:

1. **Extracting User Requests**: Identify the keywords that reflect the user’s request in the natural-language question.
2. **Converting User Request to Variables**: Map the extracted keywords to the variable names in RDF-config.
3. **Extracting User-Specified Condition**: Identify the filter conditions in the question to retrieve the desired data.
4. **Converting Conditions to Parameters**: Map each condition to its corresponding variable name and create the parameters (variable-value pairs) required for RDF-config input.

These four steps are laid out in the prompt. Implementation details are provided in Supplementary Section S.3.

## Benchmark creation

We constructed benchmark datasets for three databases: UniProt (Jain et al. 2009), Rhea (Bansal et al. 2022), and Bgee (Bastian et al. 2021). Details of these databases can be found in Supplementary Section S.1. We used the RDF-config schema files available at the time of our experiments for UniProt, Rhea, and Bgee. While the full schema files were used for Rhea and Bgee, a subset of the UniProt schema file was used in this study. To keep the number of variables provided to the baseline prompt-tuning pipeline manageable, we used a pruned version that retains the 30 most frequent, entity-rich UniProt classes and their associated properties. More details are provided in Supplementary Section S.2. These datasets were used to evaluate performance on individual databases. We also created a dataset to evaluate the performance of a cross-database search. The dataset consists of questions involving UniProt and Bgee. For the cross-database search, we used a SPARQL endpoint provided by RDF Portal (Kawashima et al. 2018), which hosts multiple RDF datasets, including UniProt and Bgee. Each benchmark dataset consists of questions and their corresponding SPARQL queries. We first created expert-made question-query pairs (original datasets), and then generated paraphrased versions of these questions in order to increase the number of questions (paraphrased datasets). Table 1 shows the number of questions included in each dataset. To properly evaluate the performance of the proposed method, it is essential that the questions are scientifically meaningful and answerable using the target database. To ensure this, experts manually created questions by referencing the target databases and their schemas. For the cross-database dataset, the questions were designed to require information from both UniProt and Bgee. After creating the questions, the experts used the RDF-config schema to select relevant variables and assign binding values. Based on these inputs, the corresponding SPARQL queries were then generated using RDF-config and treated as the ground truth SPARQL queries. We used RDF-config rather than writing the queries manually from scratch, because this approach allowed us to use the manually selected variables and parameters as the ground truth for evaluating the accuracy of variable and value selection. For relatively straightforward questions, the queries generated by RDF-config were nearly identical to those written manually. It should be noted that we evaluated query generation performance not based on the textual similarity of SPARQL queries, but on the equivalence of their results. Therefore, we considered that the use of RDF-config did not compromise the fairness of the evaluation. The number of questions for each database in these benchmarks is summarized in Table 1. The paraphrased datasets were generated using an LLM, which can successfully produce alternative expressions with the same scientific meaning. For example, it paraphrased the original question, “What is the UniProt ID for the protein associated with the ACE2 gene?” into “Please tell me the UniProt database identifier for the protein encoded by the ACE2 gene.”

**Table 1:** The number of questions included in the benchmark.

	UniProt	Rhea	Bgee	UniProt&Bgee
Original	50	21	21	17
Paraphrased	100	42	42	34
Total	150	63	63	51

The procedure of the LLM in our proposed method is to select a variable from the list created from the schema and to generate a value from a given question text. While the former merely involves selecting an appropriate variable name, the latter requires generating a binding value that exactly matches the corresponding term in the target database. This requires the normalization of named entities, such as recognizing *Mus musculus* and mouse as referring to the same entity. This problem is known as named entity recognition (NER) and named entity normalization (NEN), and has been extensively studied (Song et al. 2021). However, since NER and NEN are beyond the scope of this study, we assumed that the binding values appearing in the question text matched the entity names used in the target database. We noted that NER and NEN were necessary for practical use, and we employed a state-of-the-art tool HunFlair2 (Sänger et al. 2024) in the implementation of our proof-of-concept chatbot system. Details are described in the Application section. We also note that separating the NER task from the rest of the SPARQL generation is an approach found in a previous study (Chen et al. 2025), which supports our decision to evaluate Text-to-SPARQL models on benchmarks where entity recognition is handled as a separate step. This separation avoids introducing additional degrees of freedom (e.g. the choice and tuning of NER/NEN modules) that would otherwise make baseline comparisons less controlled.

## Experimental setup

### Evaluation methods

**Query Generation:** In previous studies (Rony et al. 2022, Rangel Reyes et al. 2024), the quality of generated queries has been evaluated based on their textual similarity to ground truth SPARQL queries, using metrics such as BLEU. However, textual similarity does not always reflect the syntactic and structural characteristics of SPARQL. A single natural language question can correspond to multiple valid query formulations, and even minor typographical errors in a SPARQL query can lead to substantially different execution results. To address these issues, we adopt an execution-based evaluation, similar to the approach used in Knowledge Graph Question Answering (KGQA) benchmarks where both the generated and ground truth queries are executed and their results are compared. Specifically, we assess the similarity between result tables as follows (Tables 2 and 3).

**Table 2:** Jaccard scores between ground truth and generated queries.

	UniProt	Rhea	Bgee	UniProt&Bgee
Proposed Method	**0.601**	**0.834**	**0.623**	**0.569**
Prompt Tuning	0.209	0.268	0.206	0.000
Fine Tuning	0.143	0.558	0.381	0.048

**Table 3:** Jaccard scores between ground truth and generated queries under different settings for variables and parameters selection.

	UniProt	Rhea	Bgee	UniProt&Bgee
VwE	**0.601**	**0.834**	**0.623**	**0.569**
VwoE	0.517	0.774	0.577	0.498
VinS	0.272	0.351	0.588	0.374

VinS: Variable in Schema; VwE: Variable with Explanation; VwoE: Variable without Explanation.

**Figure 5:**
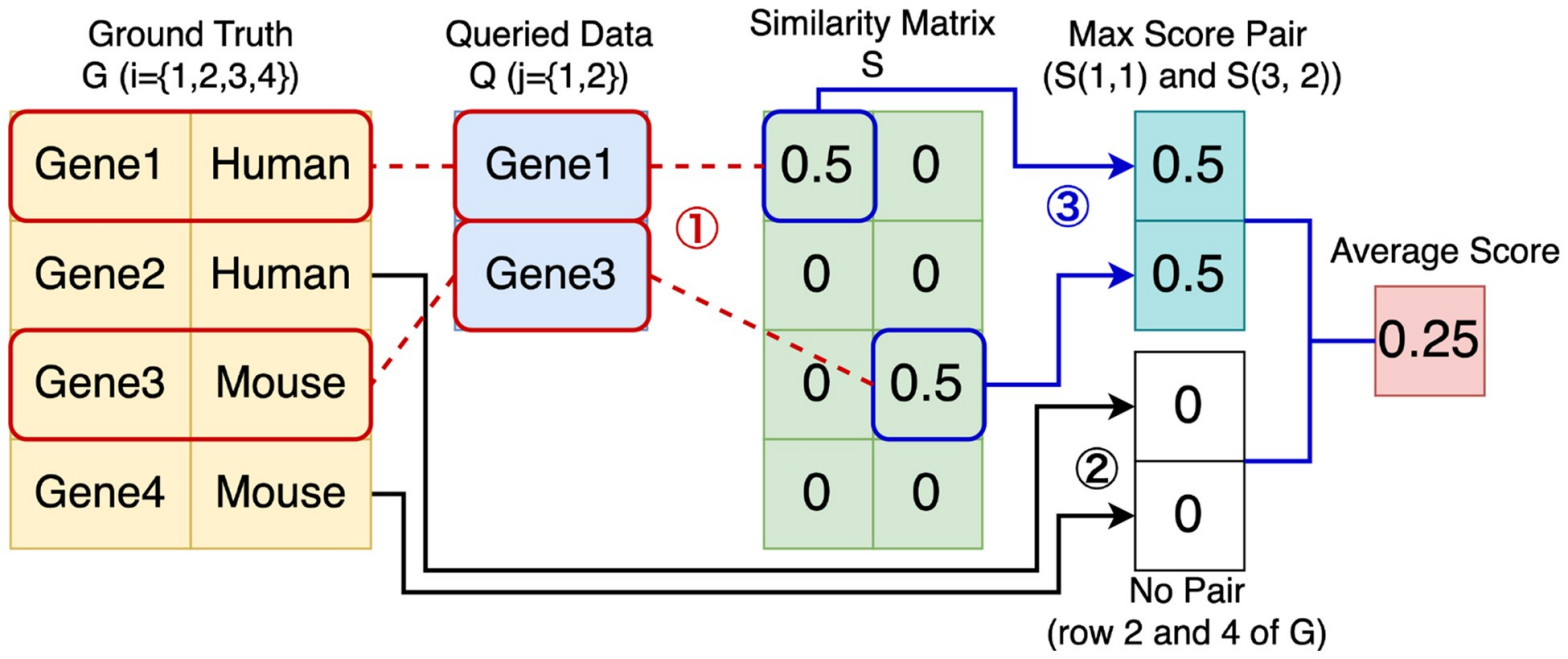
Example of average Jaccard coefficient calculation.

The result of a SPARQL query is a table, where each row contains a set of values. To measure the similarity between two rows (i.e. two sets of values), we use the Jaccard coefficient. Since the correct matching between rows in the result tables is unknown, we compute the Jaccard coefficient for all possible row pairs and solve a maximum weight matching problem to find the pairing that maximizes the total similarity. More precisely, let the result of the ground truth query be represented as a $N_{g}$ by $L_{g}$ matrix *G*, and that of the generated query as a $N_{q}$ by $L_{q}$ matrix *Q*. We compute the Jaccard coefficient between every pair of rows $G_{i,\cdot }$ and $Q_{j,\cdot }$, resulting in a $N_{g}$ by $N_{q}$ similarity matrix *S*, where $S_{i,j}$ denotes the Jaccard coefficient between row *i* of *G* and row *j* of *Q*. We then obtain the optimal set of matches $M\subseteq \{(i,j)|i\in [1,N_{g}],j\in [1,N_{q}]\}$ by solving the maximum weight matching problem on *S*. The final similarity score is calculated as $1/\max(|G|,|Q|)\cdot \sum_{(i,j)\in M}S_{i,j}$, where normalization by max(|G|,|Q|) penalizes unmatched rows. An example of the calculation is shown in Fig. 5. We found that Jaccard coefficient-based evaluation gives a zero score even when results are semantically equivalent, such as returning Mouse instead of *Mus musculus*. Although such cases were not reflected in the score in this study, applying NEN before evaluation could address the issue.

**Variable Selection and Parameter Extraction:** Since the quality of generation is influenced by how schema information is presented, we compared the accuracy of variables and parameters extraction for three settings: (i) the proposed method, where both variables and their explanations are provided (VwE), (ii) a variant that provides only the list of variables without explanations using the same prompt (VwoE), and (iii) a method that includes the whole RDF-config schema file in the prompt (VinS). To evaluate these methods, we computed the Jaccard coefficient between the predicted and ground truth sets of variables and parameters, respectively, for each question.

### Baseline methods

We compare the performance of the proposed method with two baseline approaches in which the LLM directly generates a SPARQL query: one based on fine-tuning and the other on prompt-tuning.

In the fine-tuning approach, which has also been employed in previous studies (Rony et al. 2022, Rangel Reyes et al. 2024), the model is trained on ground truth question-query pairs and generates a SPARQL query directly from a given question. We used the model gpt-4o-mini-2024–07-18 via the OpenAI API. The training data consisted of three components: a System Prompt providing task instructions, a User Message containing the input question, and an Assistant Message containing the corresponding SPARQL query. To evaluate the fine-tuning baseline robustly, we adopt a leave-one-set-out protocol. Our benchmark is organized into sets of question variants per underlying SPARQL structure: one original English question and two English paraphrases (three questions per set). In each run, we hold out one entire set as the test set and use all remaining sets as training data to fine-tune the model, repeating this procedure across all sets and reporting the average.

In the prompt-tuning approach, the database’s schema is included directly within the prompt (Fig. 4, available as supplementary data at *Bioinformatics* online), enabling the model to access the structural information required for SPARQL query generation. This baseline requires no additional training; we evaluate it on the entire benchmark and compute the score in the same manner as our proposed method.

## Results

### Results of SPARQL query generation

We tested our method and two baseline methods, fine-tuning and prompt-tuning, on four datasets (UniProt, Rhea, Bgee, and UniProt&Bgee). In each experiment, only a corresponding variable list, question-query pairs, or full schema was given to our method, the fine-tuning method, and the prompt-tuning method, respectively. That is, when evaluation was conducted on the UniProt dataset, only the variable list of UniProt was given to our method. Table 2 compares the average Jaccard coefficients of our method and two baseline methods. As shown in the table, our method outperformed the baseline methods for all four datasets. Within our method, a decline in performance was observed when multiple databases were combined (i.e. UniProt&Bgee), likely because the LLM needed to extract more variables and parameters from a larger pool. For instance, the average number of variables per question is 1.22 for UniProt, whereas it increases to 2.12 for UniProt&Bgee.

### Results of variable selection and value extraction

**Figure 6:**
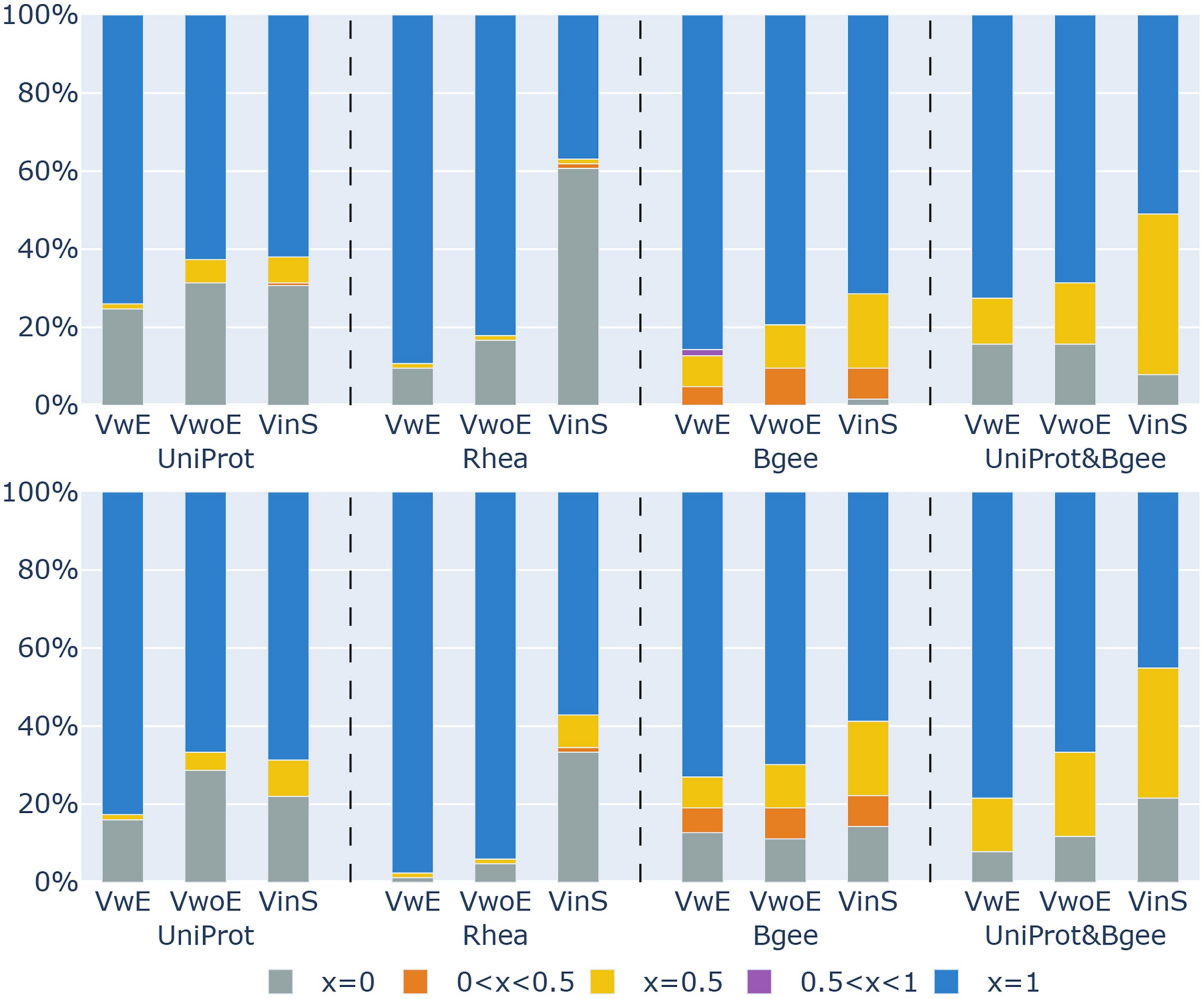
Distribution of the Jaccard scores comparing ground truth and generated variables (top) and values (bottom) across five ranges (x=0,0<x<0.5,x=0.5,0.5<x<1.0,x=1.0) under three input settings: Proposed Method (VwE), Without Explanation (VwoE), and RDF-config Schema (VinS).

Figure 6 shows the distribution of the Jaccard coefficient between the ground truth and the generated variable and value sets for each question, under three different settings (VwE, VwoE and VinS) as described in the Experimental Setup section. For all datasets, the proposed method, which uses both variables and their explanations, achieved the highest accuracy, and the generated variable and value sets were identical to the ground truth in more than 80% of the questions for the Rhea datasets. While the method that included the whole RDF-config schema file yielded the lowest accuracy, the method using only the list of variables (without explanations) performed much better, achieving accuracy slightly lower than the proposed method. This is likely because the variable names were already sufficiently descriptive. These results suggest that presenting selectable options with concise explanations, rather than structural information, is a more effective approach for variable selection and value extraction. For detailed examples of cases where the LLM in our proposed VwE method failed in variable extraction and value retrieval, see Table 5, available as supplementary data at *Bioinformatics* online. Since being able to generate queries from non-English questions is a practical advantage, we conducted a preliminary cross-lingual evaluation using Japanese translations of the English benchmark questions. We observed accuracy comparable to the English version in most settings (see Sections 1 and 4, available as supplementary data at *Bioinformatics* online).

### Hallucinations in extraction task

In the proposed method, failure cases are sometimes caused by hallucinations during parameter extraction. Since the benchmark questions contain text that exactly matches the terms used in the database, the required task is to copy that part without modifying the original text and concatenate it with the selected variable. Although in most cases the LLM successfully performs this copying, there are cases where this straightforward extraction fails. For example, in the Rhea dataset, given the question “angiotensin II + H2O = angiotensin-(1–7) + L-phenylalanine,” the LLM extracted it as “angiotensin II + H2O =>angiotensin-(1–7) + L-phenylalanine.” This introduced a discrepancy in operators (“=” and “=>”), which hindered correct database retrieval. Another failure case that is difficult to handle in the current method occurs in the Bgee dataset, where the LLM extracts “breast cells” from the question, while the term used in the database is “breast.” This issue could be resolved by applying NEN prior to the extraction process. Detailed examples of hallucination are shown in Supplementary Section S.5.

## Application

To showcase its real-world applicability, we developed a proof-of-concept chatbot using the proposed method. As noted in the Benchmark Creation section, real-world use requires normalizing user questions via NER and NEN to match database terms. Although the proposed method does not include this step, we applied HunFlair2 in the chatbot to map colloquial terms to standardized identifiers. For example, HunFlair2 identifies “Mouse” in the question “What is the expression level of the APOC2 gene in each tissue of Mouse?” and converts it to “10090”, corresponding to the NCBI taxonomy. The NEN layer is modular and can be readily replaced with tools other than HunFlair2.

**Figure 7:**
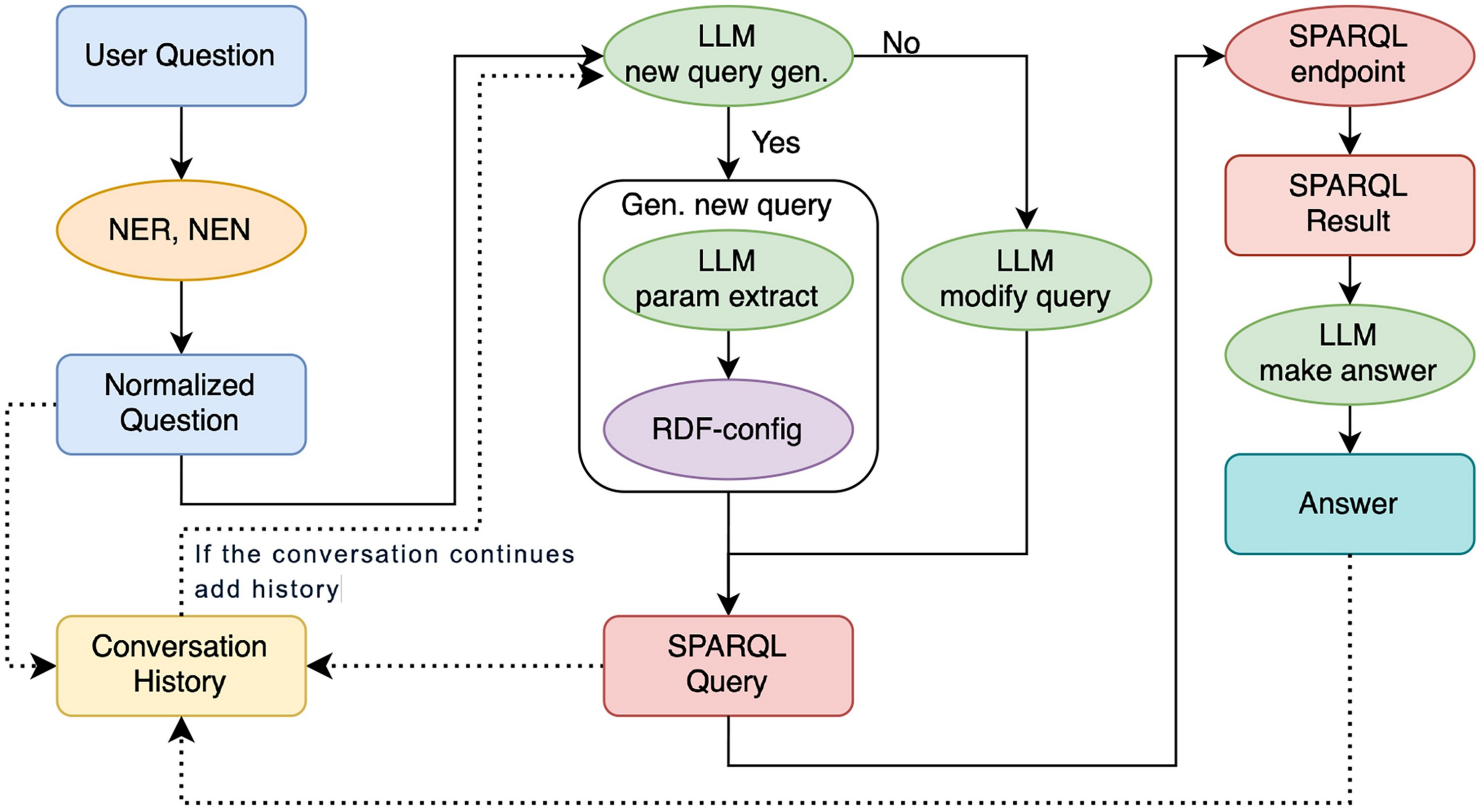
This figure illustrates the architecture of chatbot. First, a user question is normalized via NER/NEN. Then LLM decides whether to generate a new query or edit an existing one based on conversation history. A SPARQL query is created and executed; endpoint results ground the final answer. All artifacts are stored in the conversation history for reuse in subsequent turns.

Figure 7 illustrates the architecture of the chatbot system. The scenario of this chatbot proceeds as follows. First, when a user query is given, the query is first normalized by HunFlair2. Next, the normalized question is converted into a SPARQL query using the proposed method. The SPARQL query is then executed, and the result is formatted into a natural language response by the LLM. When the user provides a follow-up question, the chatbot retains the previous question and its response by including the history of questions and answers in the prompt. Therefore, if the new question is related to the previous one, the LLM refers to the previously generated SPARQL query and appropriately modifies it. For example, if the initial question is “What is the UniProt ID for the protein associated with the ACE2 gene?”, the corresponding SPARQL query is a SELECT statement. When the user then asks, “Count up the proteins of the ACE2 gene,” the LLM modifies the SELECT statement into a COUNT statement, thereby generating an appropriate response. RDF-config is limited to generating SELECT statements, but this can be addressed through such post-processing of the query. With this post-processing, our chatbot supports various SPARQL functionalities including COUNT, ASK, FILTER, OPTIONAL, UNION, and REGEX operations. Since the initial SPARQL queries are syntactically correct as they are generated by RDF-config, the post-processed queries that include these advanced operations are also likely to remain syntactically valid.

**Figure 8:**
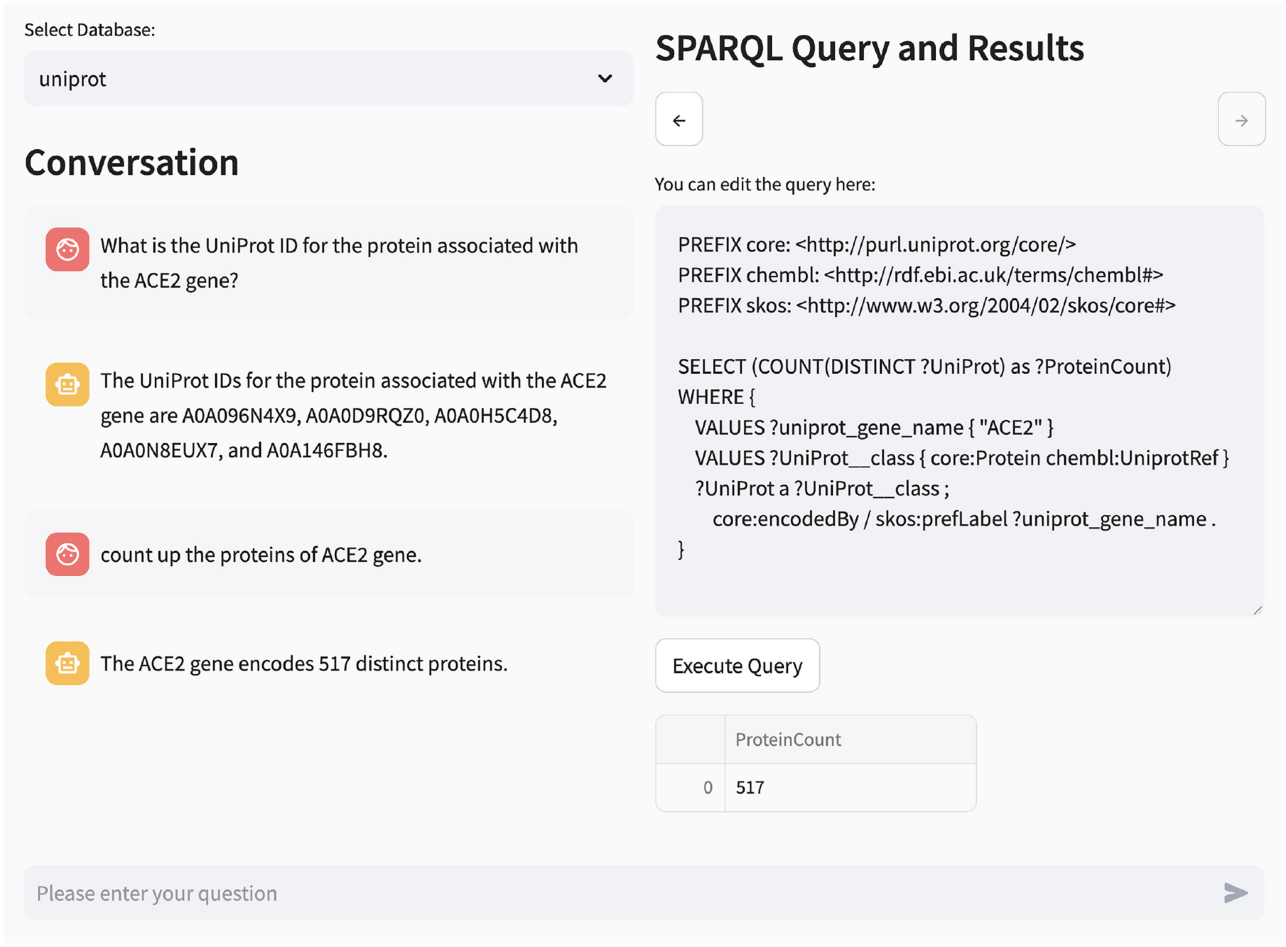
Example of chatbot-user interaction.

To maintain transparency, our system displays both the generated queries and their execution results, allowing users to check the accuracy of responses and identify potential LLM hallucinations. This approach to evidence presentation distinguishes our implementation from existing tools in the field. Figure 8 shows the chatbot’s response to the ACE2-related follow-up question introduced earlier. The interface displays both the modified SPARQL query and its execution result (447 proteins).

## Conclusion and future work

We proposed a novel approach for SPARQL query generation that combines LLM-based word extraction with a schema-based SPARQL query builder. A key advantage of our method is that it does not require question-query training datasets, whereas previous LLM-based approaches depend on large-scale annotated data. In our framework, the role of the LLM is limited to selecting and extracting relevant variables and parameters from a predefined list and the input question, which is significantly simpler than generating an entire SPARQL query. The query builder RDF-config used in our framework can automatically generate a SPARQL query from a set of variables and parameters, where the variables are defined in advance based on the structure of the target database. While we used RDF-config, other schema modelling languages, such as LinkML (https://linkml.io/linkml/; accessed in August 2025), might also be applicable with further adaptation. The advantage of using RDF-config is that it already provides schemas for widely used databases. Moreover, defining a new schema for an additional database is straightforward. Experiments using expert-curated benchmarks demonstrated that our method outperformed baseline methods that directly generate SPARQL queries, including both a fine-tuned model trained on question-query pairs and a prompt-tuned model using the full RDF-config schema. Although our expert-curated benchmark datasets are limited in size and while further validation using larger datasets would provide additional support, these results suggest that it is efficient to divide the overall task into two subtasks. The first subtask is to understand the question and select relevant schema terms, and the second is to generate a syntactically correct SPARQL query from the selected terms. Assigning the first task to an LLM and the second to a rule-based query builder helps to avoid hallucination and improves accuracy. Compared with fully rule-based text-to-SPARQL pipelines, this decomposition also reduces the manual engineering effort required to achieve broad linguistic coverage. Fully rule-based approaches typically depend on handcrafted grammars or phrase-to-predicate mappings, which are costly to maintain and tend to be brittle under paraphrases and previously unseen formulations.

We also developed a proof-of-concept chatbot system to demonstrate interactive question answering. In this system, NER and NEN are performed on the user’s question during pre-processing, and a more complex query is generated by modifying the initially generated SPARQL query in the post-processing step. Through a small number of test cases, we confirmed that this chatbot was able to resolve the limitations of the proposed method; however, a more comprehensive performance evaluation is necessary. In the proposed method, only the schema of the target database is provided in the prompt. While variable selection and parameter extraction performed well for a single database, accuracy decreased in the cross-database setting involving two databases, and is expected to degrade further with three or more. Automatic database selection and scaling to more databases remain future work. In addition, because an incorrect underlying graph pattern precludes downstream SPARQL functionality, we validated the structural correctness of generated queries with SELECT queries in our benchmark. However, real-world applications often require more advanced operations such as aggregation. Building on the post-processing strategy described in the Application section, we will formalize and systematically evaluate extensions beyond SELECT, together with expanding the benchmark. Potential improvement in variable selection and parameter extraction could be achieved by refining the existing variable descriptions. Since the LLM functions as a black box, further systematic investigation is needed to determine which factors strongly influence performance, and the results could guide database designers in preparing LLM-oriented documentation.

## Supplementary Material

btag174_Supplementary_Data

## Data Availability

The data and code underlying this article are available in the GitHub repository at https://github.com/scott2121/sparql_query_generator and archived in Zenodo at https://doi.org/10.5281/zenodo.18539213.
