# Supplementary material for: Accurate SPARQL generation via in-context learning and schema-based query construction
Source: Bioinformatics. 2026 Apr 8;42(5):btag174. doi: 10.1093/bioinformatics/btag174 (PMC13163174; doi:10.1093/bioinformatics/btag174)
Supplement: btag174_Supplementary_Data [file btag174_supplementary_data.pdf]

Supplementary materials for "Accurate SPARQL Generation via In-Context Learning and Schema-based Query Construction"

Authors: Hikaru Nagazumi, Yuki Moriya, Shuichi Kawashima, Toshiaki Katayama, Kana Shimizu

S.1 Creation of Question–SPARQL Pairs for the Benchmark

We selected the following databases and utilized their endpoints for our study.

- **UniProt**[1] is a comprehensive database of protein sequences annotated with functional information, integrating data from literature, automated systems, and community contributions. SPARQL endpoint: https://sparql.uniprot.org/sparql.
- **Rhea**[2] is a curated knowledgebase of biochemical reactions based on the ChEBI ontology, providing comprehensive reaction data for studying and engineering enzymes. It serves as a reference vocabulary for enzyme annotation in UniProtKB and supports the exploration of metabolic systems. SPARQL endpoint: https://sparql.rhea-db.org/sparql.
- **Bgee**[3] is a database that integrates curated healthy gene expression data from multiple animal species using various data types to provide a reference for normal gene expression patterns across species. SPARQL endpoint: https://www.bgee.org/sparql.

For integrated searches, we employed RDF-portal[4] (https://rdfportal.org/sib/sparql), which integrates multiple databases into a single SPARQL endpoint, to execute queries across UniProt and Bgee.

S.1.1 Benchmark Construction for a Single Database

The benchmark dataset is built through a structured workflow that combines human experts with Large Language Models (LLMs). The detailed steps and illustrative examples are given below.

1. **Question Generation by Domain Experts**
   After selecting the target database, experts then formulate natural-language questions for the database.
   - Example (UniProt): "What is the EC number for the ACE2 gene?"
2. **SPARQL Queries Generation with RDF-config**
   For each question, experts map key terms to variables defined in the RDF-config schema and execute RDF-config to generate the corresponding SPARQL query. The expert then verifies that the generated query faithfully reflects the original question.
   - Inputs for RDF-config Based on Questions
     - **Variables (output)**: `?uniprot_recommended_ec` — the EC number(s) to be returned.
     - **Parameters (filter)**: `?uniprot_gene_name` fixed to "ACE2" — the gene name used to restrict the result set.

- Output Query from RDF-config
  The generated SPARQL query is shown in Figure S.1.

```
PREFIX core: <http://purl.uniprot.org/core/>
PREFIX chembl: <http://rdf.ebi.ac.uk/terms/chembl#>
PREFIX skos: <http://www.w3.org/2004/02/skos/core#>

SELECT DISTINCT ?uniprot_recommended_ec
WHERE {
    VALUES ?uniprot_gene_name { "ACE2" }
    VALUES ?UniProt__class { core:Protein chembl:UniprotRef }
    ?UniProt a ?UniProt__class ;
        core:recommendedName / core:ecName ?uniprot_recommended_ec ;
        core:encodedBy / skos:prefLabel ?uniprot_gene_name .
}
LIMIT 100
```

Figure S.1: SPARQL Generated by RDF-config

3. **Converting Questions into Templates**
   To facilitate later substitution and prevent lexical variation during LLM-based paraphrasing, database-specific words in each question are replaced with variables enclosed in curly braces (e.g., `{uniprot_gene_name}`).
   - **Original:** "What is the EC number for the ACE2 gene?"
   - **Template:** "What is the EC number for the {uniprot_gene_name} gene?"
4. **Paraphrase Generation by LLM**
   LLMs are used to rephrase each templated question, producing diverse natural-language variants.
   - **Template:** "What is the EC number for the {uniprot_gene_name} gene?"
   - **Paraphrase:** "I want to know the Enzyme Commission number for the enzyme coded by the {uniprot_gene_name} gene."
5. **Translation by LLM**
   LLMs translate questions into additional languages, in this case into Japanese.
6. **Validation and Quality Control by Experts**
   Experts review all generated paraphrases and translations for accuracy and clarity to the intended meaning. Any problematic instances are corrected or discarded.

This comprehensive process ensures that the benchmark dataset accurately captures variability in question phrasing and structure, while maintaining consistency and accuracy in query generation.

S.1.2 Creation of Benchmark for cross-database search

Below, we present the procedure for creating a benchmark for cross-database search.

1. Select a pair of questions from each database. In the example below, both questions refer to the gene "ACE2":
   - **UniProt:** "What is the UniProt ID for the protein associated with the ACE2 gene?"
   - **Bgee:** "What is the expression level of the ACE2 gene?"
2. Combine these into a single cross-database query:

   "What is the expression level of the ACE2 gene, and what is its UniProt ID?"

   This ensures that both databases must be consulted to answer the question.
3. From this point onward, proceed with the steps described in Section S.1.1, starting at Step 3.

S.1.3 Benchmark Examples

The Table S.1 below presents example benchmark question–SPARQL query pairs in three columns: "Question Type," indicating whether each row is the original English question, one of its English paraphrases, or the Japanese translation; "Question," containing the question text in the corresponding language; and "Query," containing the SPARQL query that corresponds to that question.

<table>
<tr><th>Question Type</th><th>Question</th><th>Query</th></tr>
<tr><td>Original</td><td>Please tell me about the annotations regarding the protein function of the ACE2 gene.</td><td rowspan="6">PREFIX core: <http://purl.uniprot.org/core/>
PREFIX chembl: <http://rdf.ebi.ac.uk/terms/chembl#>
PREFIX rdfs: <http://www.w3.org/2000/01/rdf-schema#>
PREFIX skos: <http://www.w3.org/2004/02/skos/core#>
SELECT DISTINCT ?function_annotation_desc
WHERE {
  VALUES ?uniprot_gene_name { "ACE2" }
  VALUES ?UniProt__class { core:Protein chembl:UniprotRef }
  ?UniProt a ?UniProt__class ;
    core:encodedBy / skos:prefLabel ?uniprot_gene_name .
  OPTIONAL {
    ?UniProt core:annotation ?FunctionAnnotation .
    ?FunctionAnnotation a core:Function_Annotation ;
      rdfs:comment ?function_annotation_desc .
  }
}</td></tr>
<tr><td>Paraphrased1</td><td>Explain the biological role of the protein encoded by the ACE2 gene.</td></tr>
<tr><td>Paraphrased2</td><td>Please share what is known about the known functions and activities of the ACE2 protein.</td></tr>
<tr><td>Translated Original</td><td>ACE2遺伝子のタンパク質機能に関する注釈を教えてください。</td></tr>
<tr><td>Translated Paraphrased1</td><td>ACE2遺伝子がコードするタンパク質の生物学的役割について説明してください。</td></tr>
<tr><td>Translated Paraphrased2</td><td>ACE2タンパク質の既知の機能や活性について、わかっていることを教えてください。</td></tr>
</table>

Table S.1: Queries on ACE2 protein function annotations for UniProt

S.2 RDF-config Preparation

The model.yaml files for some databases are available in config files on RDF-config GitHub repository [5]. However, for some databases, it was necessary to update the model structures to reflect recent data revisions. Additionally, since there was no existing model.yaml for Bgee, we created a new configuration from scratch and contributed it to the RDF-config repository.

For Rhea, we used the existing RDF-config schema file provided in the repository, without any additional pruning. For Bgee, we used the RDF-config schema file that we newly created and contributed to the repository, likewise without further pruning. These RDF-config models were originally curated by domain experts and already represent subgraphs of the full RDF models, focusing on entities and relations that are relevant for typical queries in each database. In contrast, the available RDF-config schema for UniProt (model.yaml) is substantially larger, at about 1,200 lines. When we included this full file directly in the LLM prompt, we observed that it consumed a large portion of the context window and degraded the effectiveness of our prompt-tuning pipeline. To keep the prompt size manageable while still covering frequently used parts of the UniProt graph, we expanded our frequency-based subset to the top 30 UniProt classes (roughly 350 lines) and their associated properties. This heuristic is a practical way to preserve the most commonly used portions of the UniProt schema under current context-length constraints. Currently, our system does not implement query-dependent schema selection for the LLM prompt, and we therefore rely on this fixed subset for UniProt. As future work, we plan to add a schema-selection component that automatically narrows the RDF-config model per query, enabling us to expose a larger fraction of the UniProt schema without overloading the LLM context.

Because an RDF-config model.yaml simply encodes the existing database schema, creating a new one requires only modest effort for practitioners who are already familiar with the database. For small databases with only a few classes (for example, gene and variant), this can often be done within an hour. For very complex databases, where dozens of classes each have many attributes, the process may take several days to weeks. Additionally, a tool called Shexer has been developed that automatically generates a template `model.yaml` for use with RDF-config by inferring a schema from RDF data. Because only minor adjustments to the vocabulary and naming conventions are needed on top of the template, the time required to produce the `model.yaml` can be substantially reduced. These model.yaml files have been designed to generate SPARQL queries for extracting variable-specific values from the databases.

```
- UniProt up:Q9BYF1:
  - a:
    - core:Protein
  - core:recommendedName:
    - []:
      - a: core:Structured_Name
      - core:fullName:
        - uniprot_recommended_name: "Angiotensin-converting␣enzyme␣2"
      - core:shortName:
        - uniprot_recommended_short_name: "hVps37A"
      - core:ecName:
        - uniprot_recommended_ec: "3.4.17.23"
  - core:encodedBy:
    - []:
      - a: core:Gene
      - skos:prefLabel:
        - uniprot_gene_name: "ACE2"
      - skos:altLabel:
        - uniprot_gene_alt_name: "UNCX4.1"
      - core:orfName:
        - uniprot_orf_name: "UNQ868/PRO1885"
```

Figure S.2: A snippet of the UniProt model.yaml

Typically, RDF-config is designed to handle a single database, but by combining multiple model.yaml files, it can support cross-database searches. In this study, we created a unified configuration file by merging the model.yaml files of UniProt and Bgee to enable cross-database searches between these two databases. This allows us to execute common queries across both databases and perform cross-database searches. The fourth line in the Bgee model.yaml example figure S.3 refers to UniProt; if you align that reference with the terminology used in the UniProt model.yaml (figure S.2), rdf-config can join the two databases at runtime.

```
- Gene oma:GENE_ENSG00000216412:
  - a: orth:Gene
  - lscr:xrefUniprot:
    - gene_uniprot_id: UniProt
  - lscr:xrefNCBIGene:
    - gene_ncbigene_id: ncbigene:118230125
  - lscr:xrefEnsemblGene:
    - gene_ensembl_id: ensg:ENSG00000130208
```

Figure S.3: A snippet of the Bgee model.yaml for cross-database search

S.3 Proposed Method Implementation Details

RDF-config was executed as a Ruby command using the following format:

```
bundle exec rdf-config --config config/{database} --sparql {id}
```

where database denotes the database name (e.g., uniprot) and id refers to the question identifier. The generated SPARQL queries were then processed using Python 3.8, with SPARQLWrapper (v2.0.0) alongside rdflib (v7.1.1). For non–fine-tuning experiments, the OpenAI API was called with gpt-4-1106-preview model, and all other parameters at their defaults. Fine-tuning experiments used the gpt-4o-mini-2024-07-18 model with default hyperparameters.

S.3.1 Baseline method based on prompt tuning

Figure S.4 shows the prompt used for the prompt-tuning method. The placeholders {user_question}, {prefix_str}, and {model_yaml} are replaced with the natural-language question, the required PREFIX list, and the YAML schema, respectively.

Figure S.4: Prompt used for the Prompt Tuning method.

```
f"""
Create a SPARQL query to retrieve values from the database for the user question provided below:
User Question: '{user_question}'

Note: Database structure information is provided in YAML format. Use this information effectively and
↪  accurately to ensure the query is feasible.

Prefixes:
{prefix_str}

Database Structure (YAML):
{model_yaml}

[INSTRUCTIONS]:
- Create a simple SPARQL query using the YAML database structure provided to answer the user question.
- Use only URI references that are explicitly listed in the YAML schema. Do not introduce any elements that
↪  are not part of the actual schema.
- Apply the FILTER clause judiciously to refine your results. Ensure that your query effectively uses the
↪  attributes and relationships detailed in the YAML without omitting valid data.
- Do not modify the values from the database using BIND, STRAFTER, or other operations, such as trimming text.
↪  The SPARQL output for the question should directly return values from the database.

---
Now Create a SPARQL query to retrieve values from the database for the user question.
First, explain the content of the SPARQL query you need to write,
Second, check if it follows the [INSTRUCTIONS].
Finally, create the SPARQL query.
"""
```

S.4 Score Differences Between English and Japanese Questions

To evaluate performance differences due to question language, we created a set of Japanese questions by translating the original English ones (see Table S.2 for the number of questions per benchmark). We emphasize that the experiments reported in the main text are conducted solely in English, i.e., models are trained and evaluated using English questions only. In this supplementary section, we extend the evaluation to both English and Japanese queries to quantify language-dependent score changes. Importantly, for Fine-Tuning, we also examine the effect of adding Japanese questions during training while keeping the evaluation protocol unchanged. Specifically, we evaluate on the English test set (as in the main text) and compare two fine-tuning setups—English-only versus English+Japanese training (Table S.3). We observe that including Japanese questions in the fine-tuning data tends to yield slightly higher scores even on English evaluation in our setting. Based on this observation, unless otherwise noted, we use the English+Japanese fine-tuning setting in the following experiments. This section analyzes the Jaccard scores of our proposed method (VwE), proposed method without variable explanations (VwoE), proposed method with RDF-config schema (VinS), and baseline methods (Prompt Tuning and Fine-Tuning) across both English and Japanese queries. The full results are presented in Table S.4.

Table S.2: The number of questions included in the benchmark

	UniProt	Rhea	Bgee	UniProt&Bgee
Original	50	21	21	17
Paraphrased	100	42	42	34
English Total	150	63	63	51
Japanese Total	150	63	63	51
Total	300	168	126	102

Table S.3: Effect of incorporating Japanese questions into FT baseline on English evaluation.

Training data	UniProt	Rhea	Bgee	UniProt & Bgee
English-only	0.143	0.558	0.381	0.048
English+Japanese	0.297	0.623	0.413	0.068

Table S.4: Jaccard Score Comparison Between English and Japanese Questions for Each Method

Method	Language	UniProt	Rhea	Bgee	UniProt & Bgee
Proposed Method (VwE)	English	**0.601**	**0.834**	0.623	**0.569**
	Japanese	0.575	0.821	**0.635**	0.482
VwoE	English	0.517	0.774	0.577	0.498
	Japanese	0.489	0.821	0.624	0.560
VinS	English	0.272	0.351	0.588	0.374
	Japanese	0.272	0.316	0.563	0.244
PT (Baseline)	English	0.209	0.268	0.206	0.0
	Japanese	0.146	0.217	0.165	0.0
FT (Baseline)	English	0.297	0.623	0.413	0.068
	Japanese	0.232	0.625	0.365	0.098

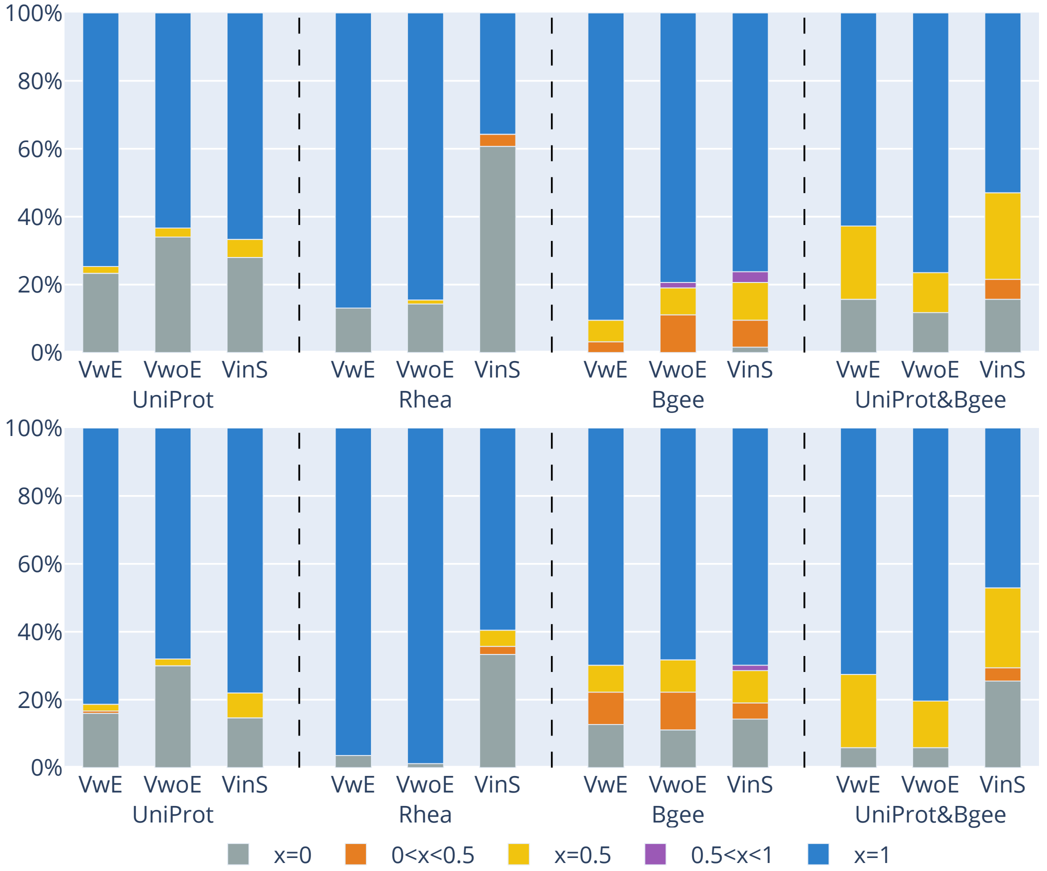

Figure S.5: Distribution of the Jaccard scores for Japanese questions, comparing ground-truth and generated variables (top) and values (bottom) across five ranges ($x = 0, 0 < x < 0.5, x = 0.5, 0.5 < x < 1.0, x = 1.0$) under three input settings: Proposed Method (VwE), Without Explanation (VwoE), and RDF-config Schema (VinS).

S.4.1 Performance of the Proposed Method (VwE)

For single-database queries (UniProt, Rhea, and Bgee), our proposed method (VwE) demonstrated that performance was largely comparable between English and Japanese questions, with only negligible score differences. This suggests that the method is robust to linguistic variations in simpler search tasks.

However, a notable divergence was observed in the more complex cross-database search (UniProt & Bgee). In this scenario, English questions achieved a Jaccard score of 0.569, which was 0.087 points higher than the 0.482 scored by Japanese questions. We attribute this performance gap to the supplementary variable descriptions provided to the model, which were written exclusively in English. As the complexity and number of variables increase in cross-database queries, we hypothesize that these English-language descriptions provided a significant advantage for interpreting and processing the English questions, creating a linguistic bias.

S.4.2 Analysis with Comparative and Baseline Methods

This hypothesis is further supported by the results from the comparative and baseline methods, which reveal how different types of information influence performance across languages.

First, the VwoE method, which omits the detailed variable explanations, showed a reversed trend. In this configuration, Japanese questions outperformed English ones in several cases, most notably in the cross-database search (0.560 for Japanese vs. 0.498 for English). This suggests that without the English-language aid, the model's inherent processing capabilities or the nature of the Japanese questions can lead to superior performance.

A different pattern emerges with the VinS method, which uses only schema information without detailed explanations. Here, English consistently outperformed Japanese across all databases, with the most significant gap again appearing in the cross-database search (0.374 for English vs. 0.244 for Japanese). This suggests that providing only structural schema information, which is written in English, creates a closer match with English questions.

Figure S.5 visualizes the Jaccard scores for Japanese questions (see Figure 6 for English). It reveals a key performance inversion in the cross-database search: VwoE outperforms VwE for Japanese questions, which is the opposite of the English results. This strongly suggests that English explanations are not universally beneficial and can impair performance for other languages.

S.4.3 Discussion and Future Work

Our analysis indicates that performance in complex, multi-database queries is highly sensitive to the language of the supplementary information provided. The English-only variable descriptions boosted scores for English questions but created a performance bottleneck for Japanese ones. Future work should validate this finding by conducting experiments using Japanese-language descriptions.

S.5 Hallucinations in Parameters Extraction

Table S.5 presents concrete examples of hallucinations that occur when our proposed method extracts parameters (variable-value pairs) from natural language questions in biological database contexts. The Database column identifies the specific biological database being queried, while the Question column contains the natural language query posed to the LLM. The Correct Pair column shows the expected parameters extraction that would enable proper database querying, and the LLM Extracted Pair column displays what the model actually generated. Finally, the Failure Case column categorizes the type of error that occurred.

The observed failures can be categorized into three distinct patterns, each representing different aspects of the extraction process. Value extraction hallucinations are cases where the LLM correctly identifies the appropriate variable but fails to accurately extract the corresponding value from the question text. These errors manifest as subtle modifications to the original text, such as changing "=" to "=>" in chemical equations, replacing spaces with underscores in species names like "Homo sapiens" becoming "Homo_sapiens", or expanding terms like "breast" to "breast cells". While the variable selection remains correct, these value modifications can lead to failed database queries or incorrect results.

Variable selection failures represent cases where the LLM misunderstands which database field should be populated, despite correctly identifying relevant information in the question. For instance, when asked about a specific compound identifier, the model might choose "reaction_equation" instead of the appropriate "compound_name" variable, or select "Compound" rather than the standardized "compound_chebi" field. These errors indicate misunderstanding of the database schema or question intent.

Compound errors combine both variable selection failures and value extraction hallucinations simultaneously. In these cases, the LLM not only selects an inappropriate variable but also generates a value that fits the incorrectly chosen variable rather than extracting from the original question. For example, when asked about gene expression levels for "APOC1", the model might select the unrelated "expression_condition_developmental_stage" variable and then generate the generic value "any" to match that variable type. Similarly, when querying about "Homo sapiens", the model might choose "taxonomy_common_name" instead of "taxonomy_scientific_name" and then generate "human" as a value that corresponds to the incorrectly selected variable. These compound failures involve both conceptual misunderstanding and creative generation of plausible but incorrect information.

S.6 Evaluation Against BioSODA Benchmark

S.6.1 Benchmark Dataset

We evaluated our approach using the BioSODA benchmark dataset, which provides SPARQL queries for bioinformatics databases. Specifically, we utilized 10 queries from the Bgee database component of the benchmark, available at `https://github.com/anazhaw/Bio-SODA/tree/master/Benchmarks/Bioinformatics/Bgee`.

S.6.2 Query Corrections

During our evaluation, we identified several syntactic errors in the original benchmark queries that prevented successful execution on the Bgee SPARQL endpoint (`https://www.bgee.org/sparql/`). The following corrections were necessary:

Table S.5: Examples of hallucinations in Parameters

**Database**	**Question (Add Questions)**	**Correct Parameters**	**LLM Extracted Parameters**	**Failure Case**
UniProt	What is the name of the catalytic protein associated with the reaction angiotensin II + H2O = angiotensin-(1-7) + L-phenylalanine?	rhea_label: "angiotensin II + H2O = angiotensin-(1-7) + L-phenylalanine"	rhea_label: "angiotensin II + H2O => angiotensin-(1-7) + L-phenylalanine"	Value extraction hallucination
Bgee	What are the expression levels of each gene within breast cells in Homo sapiens?	taxonomy_scientific_name: "Homo sapiens"	taxonomy_scientific_name: "Homo_sapiens"	Value extraction hallucination
Bgee	What are the expression levels of each gene within breast cells in Homo sapiens?	anatomical_entity_name: "breast"	anatomical_entity_name: "breast cells"	Value extraction hallucination
Rhea	What is the reaction equation of the reaction that includes the L-lysyl-[histone] molecule?	compound_name: "L-lysyl-[histone]"	reaction_equation: "L-lysyl-[histone]"	Variable selection failure
Rhea	Is the net charge of this obo:CHEBI_15377 molecule positive, negative, or neutral?	compound_chebi: obo:CHEBI_15377	Compound: "obo:CHEBI_15377"	Variable selection failure
Rhea	Is the chemical equation for rhea:10024 stable?	Reaction: rhea:10024	reaction_is_chemically_balanced: true	Variable selection failure and value extraction hallucination
Bgee	What are the expression levels of the APOC1 gene during different stages of development?	gene_name: "APOC1"	expression_condition_developmental_stage: "any"	Variable selection failure and value extraction hallucination
Bgee	What are the expression levels of the APOC1 gene across different tissues in Homo sapiens?	taxonomy_scientific_name: "Homo sapiens"	taxonomy_common_name: "human"	Variable selection failure and value extraction hallucination

1. **Undefined prefixes:** Queries Q6, Q7, Q8, Q9, and Q10 contained references to the undefined prefix `orth`, which caused execution failures.
2. **Syntax errors:** Query Q10 had a missing closing parenthesis that resulted in a parsing error.
3. **Variable inconsistencies:** Query Q4 contained an incorrect variable reference (`?taxon up:commonName 'fruit fly'`) where `?taxon` should have been `?species`.

Note that while queries Q4, Q5, and Q9 also contained undefined `rdfs` prefixes, these queries remained executable without modification, so we preserved them in their original form.

Experimental setup (query generation)

Beyond correcting the benchmark queries, we also detail our query generation pipeline. We first use our RDF-config–based method to synthesize a base SPARQL query for Bgee. We then submit the natural-language question together with the generated query to an LLM, which post-edits the query to align it with the question and to incorporate constructs that are not readily emitted by RDF-config alone (e.g., `FILTER` constraints and aggregations such as `COUNT`). The LLM–post-edited query is used for execution and evaluation.

S.6.3 Experimental Results

Table S.6 presents the comparative results between our approach and the ground-truth (GT) of the BioSODA benchmark queries. We compute each query's score by matching the execution results of the ground-truth query and our generated query, and measuring the agreement rate using the Jaccard score over sets of exactly matching results. The last column (BioSODA Precision) reports the precision between the query result set and the gold-standard result set defined by the benchmark, computed according to the definition of precision used in the original Bio-SODA paper. Here, precision is a measure of the agreement, for each question, between the set of result tuples returned by the SPARQL query corresponding to the top-ranked interpretation produced by Bio-SODA (Precision@1) and the gold-standard set of result tuples.

	Our Score	GT Res. Count	Our Method Res. Count	BioSODA Precision
Q1	1.000	52	52	1
Q2	0.000	34	36	0
Q3	0.500	11	11	1
Q4	0.333	17807	17807	0
Q5	0.000	427	1	1
Q6	0.000	TIMEOUT	TIMEOUT	1
Q7	0.000	TIMEOUT	TIMEOUT	0
Q8	0.000	64	TIMEOUT	1
Q9	0.000	TIMEOUT	TIMEOUT	1
Q10	0.289	336	581	1

Table S.6: Comparison results on BioSODA Bgee benchmark queries

Endpoint time limits The Bgee SPARQL endpoint imposes execution time constraints that affect evaluation. Among the ground-truth queries, Q6, Q7, and Q9 did not complete within the endpoint allocated time. In Table S.6, we denote such cases as `TIMEOUT` where applicable. These constraints highlight the practical challenges of executing complex biological queries on public endpoints.

To aid qualitative assessment, we also provide representative result snippets and the exact SPARQL used in our runs. The full result sets are large; therefore we include concise samples below, followed by the query listings (split across four tables for readability).

Interpreting the sample rows

Our automatic matching uses a Jaccard score over sets of key–value pairs; therefore semantically equivalent rows can be judged as non-matches when they differ only in identifier representation (e.g., URI vs. label/short ID). For example, in Q2 the ground-truth retrieves a gene via a URI (`Gene: http://omabrowser.org/ontology/oma#GENE_ENSCJAG00000053773`), whereas our method uses a compact identifier (`geneId: ENSCJAG00000053773`). Although both refer to the same entity, the Jaccard score treats them as different. To make such cases transparent, we present side-by-side samples of ground-truth outputs and our outputs below, and we also show the exact SPARQL queries used to generate them.

	Ground Truth sample results	Our sample results
Q1	taxon: http://purl.uniprot.org/taxonomy/7994, name: Astyanax mexicanus	taxonomy_scientific_name: Astyanax mexicanus, Taxon: http://purl.uniprot.org/taxonomy/7994
Q2	geneName: LOC100406216, geneId: ENSCJAG00000053773	Gene: http://omabrowser.org/ontology/oma#GENE_ENSCJAG00000053773
Q3	name: Xenopus laevis	taxonomy_common_name: African clawed frog, taxonomy_scientific_name: Xenopus laevis
Q4	gene: http://omabrowser.org/ontology/oma#GENE_FBgn0000003, geneName: 7SLRNA:CR32864	gene_name: 7SLRNA:CR32864, gene_identifier: FBgn0000003
Q5	anatName: blood cell	No data, because of query failure
Q8	anatEntity: http://purl.obolibrary.org/obo/GO_0005575, anatName: cellular_component	No data, due to timeout
Q10	anatEntity: http://purl.obolibrary.org/obo/GO_0005575, anatName: cellular_component	anatomical_entity_name: cellular_component

Table S.7:

SPARQL listings and failure query

In this section, we present the exact SPARQL queries executed during evaluation. Beyond the sample-level evidence of similar results, we conduct a side-by-side inspection of the generated and ground-truth queries in terms of basic graph patterns, joins, constraints, and aggregations. This analysis reveals no substantive semantic divergence in the intended result sets; most differences arise from implementation details such as URI vs. label representations and FILTER thresholds. An exception is Q5, where a hallucinated variable name in the LLM-generated query caused a failure to return any results; the erroneous portion is highlighted in red in the table.

	Ground truth	Our Method
Q1	PREFIX up:<http://purl.uniprot.org/core/><br>select * {<br>?taxon a up:Taxon.<br>?taxon up:scientificName ?name.<br>?taxon up:rank up:Species.<br>}	PREFIX genex: <http://purl.org/genex#><br>PREFIX orth: <http://purl.org/net/orth#><br>PREFIX obo: <http://purl.obolibrary.org/obo/><br>PREFIX core: <http://purl.uniprot.org/core/><br><br>SELECT DISTINCT ?taxonomy_scientific_name ?Taxon<br>WHERE { ?Taxon a core:Taxon ;<br>core:scientificName ?taxonomy_scientific_name . }
Q2	PREFIX up:<http://purl.uniprot.org/core/><br>PREFIX orth:<http://purl.org/net/orth#><br>PREFIX dcterms:<http://purl.org/dc/terms/><br>select ?geneName ?geneId {<br>?gene a orth:Gene.<br>?gene rdfs:label ?geneName.<br>?gene dcterms:identifier ?geneId.<br>?gene dcterms:description ?desc.<br>FILTER CONTAINS(?desc, 'lung')}	PREFIX genex: <http://purl.org/genex#><br>PREFIX orth: <http://purl.org/net/orth#><br>PREFIX dcterms: <http://purl.org/dc/terms/><br><br>SELECT DISTINCT ?Gene<br>WHERE { ?Gene a orth:Gene ;<br>dcterms:description ?description .<br>FILTER (CONTAINS(LCASE(STR(?description)), LCASE("lung"))) }
Q3	PREFIX up:<http://purl.uniprot.org/core/><br>PREFIX orth:<http://purl.org/net/orth#><br>PREFIX obo:<http://purl.obolibrary.org/obo/><br>select ?name {<br>?gene a orth:Gene.<br>?gene rdfs:label ?geneName.<br>?gene orth:organism ?organism. #orth v2<br>?organism obo:RO_0002162 ?taxon. #label: in taxon.<br>?taxon up:scientificName ?name.<br>FILTER (UCASE(?geneName) = UCASE('Mt-co1'))}	PREFIX genex: <http://purl.org/genex#><br>PREFIX orth: <http://purl.org/net/orth#><br>PREFIX obo: <http://purl.obolibrary.org/obo/><br>PREFIX core: <http://purl.uniprot.org/core/><br>PREFIX rdfs: <http://www.w3.org/2000/01/rdf-schema#><br><br>SELECT DISTINCT ?taxonomy_common_name ?taxonomy_scientific_name<br>WHERE { ?Gene a orth:Gene ;<br>orth:organism / obo:RO_0002162 / core:commonName ?taxonomy_common_name ;<br>orth:organism / obo:RO_0002162 / core:scientificName ?taxonomy_scientific_name ;<br>rdfs:label ?label .<br>FILTER (CONTAINS(LCASE(STR(?label)), LCASE("Mt-co1"))) }

Table S.8:

	Ground truth	Our Method
Q4	PREFIX up:<http://purl.uniprot.org/core/><br>PREFIX orth:<http://purl.org/net/orth#><br>PREFIX obo:<http://purl.obolibrary.org/obo/><br>select ?gene ?geneName {<br>?gene a orth:Gene .<br>?gene rdfs:label ?geneName .<br>?gene orth:organism ?taxon .<br>?taxon obo:RO_0002162 ?species . #in taxon property .<br>?species up:commonName 'fruit fly'.}	PREFIX genex: <http://purl.org/genex#><br>PREFIX orth: <http://purl.org/net/orth#><br>PREFIX rdfs: <http://www.w3.org/2000/01/rdf-schema#><br>PREFIX dcterms: <http://purl.org/dc/terms/><br>PREFIX obo: <http://purl.obolibrary.org/obo/><br>PREFIX core: <http://purl.uniprot.org/core/><br><br>SELECT DISTINCT ?gene_name ?gene_identifier<br>WHERE { ?Gene a orth:Gene ;<br>rdfs:label ?gene_name ;<br>dcterms:identifier ?gene_identifier ;<br>orth:organism / obo:RO_0002162 / core:commonName ?taxonomy_common_name .<br>FILTER (CONTAINS(LCASE(STR(?taxonomy_common_name)), LCASE("fruit fly")))<br>}
Q5	PREFIX up:<http://purl.uniprot.org/core/><br>PREFIX genex:<http://purl.org/genex#><br>PREFIX obo:<http://purl.obolibrary.org/obo/><br>select distinct ?anatName {<br>?cond genex:hasAnatomicalEntity ?anatEntity.<br>?anatEntity rdfs:label ?anatName.<br>?cond genex:hasDevelopmentalStage ?stage.<br>?stage rdfs:label ?stageName.<br>?cond obo:RO_0002162 ?taxon. #in taxon property.<br>?taxon up:commonName 'human'.<br>FILTER (contains(?stageName,"young") && contains(?stageName,"adult"))}	PREFIX genex: <http://purl.org/genex#><br>PREFIX rdfs: <http://www.w3.org/2000/01/rdf-schema#><br>PREFIX orth: <http://purl.org/net/orth#><br>PREFIX obo: <http://purl.obolibrary.org/obo/><br>PREFIX core: <http://purl.uniprot.org/core/><br>PREFIX efo: <http://www.ebi.ac.uk/efo/><br><br>SELECT DISTINCT ?anatomical_entity_name<br>WHERE { ?Expression a genex:Expression ;<br>genex:hasExpressionCondition / genex:hasAnatomicalEntity / rdfs:label ?anatomical_entity_name_temp ;<br>genex:hasSequenceUnit / orth:organism / obo:RO_0002162 / core:commonName ?taxonomy_common_name_temp ;<br>genex:hasExpressionCondition / genex:hasDevelopmentalStage / rdfs:label ?expression_condition_developmental_stage_temp .<br><br>FILTER (CONTAINS(LCASE(STR(?taxonomy_common_name_temp)), LCASE("human")))<br>FILTER (CONTAINS(LCASE(STR(?expression_condition_developmental_stage_temp)), LCASE("young adult"))) }

Table S.9:

	Ground truth	Our Method
Q6	PREFIX up:<http://purl.uniprot.org/core/><br>PREFIX genex:<http://purl.org/genex#><br>PREFIX obo:<http://purl.obolibrary.org/obo/><br>PREFIX orth: <http://purl.org/net/orth#><br>select distinct ?anatEntity ?anatName {<br>?seq a orth:Gene.<br>?expr genex:hasSequenceUnit ?seq.<br>?seq rdfs:label ?geneName .<br>?expr genex:hasExpressionCondition ?cond.<br>?cond genex:hasAnatomicalEntity ?anatEntity.<br>?anatEntity rdfs:label ?anatName.<br>FILTER (LCASE(?geneName) = 'apoc1')}	PREFIX genex: <http://purl.org/genex#><br>PREFIX rdfs: <http://www.w3.org/2000/01/rdf-schema#><br>PREFIX orth: <http://purl.org/net/orth#><br><br>SELECT DISTINCT ?anatomical_entity_name<br>WHERE { ?Expression a genex:Expression ;<br>genex:hasExpressionCondition / genex:hasAnatomicalEntity / rdfs:label ?anatomical_entity_name ;<br>genex:hasSequenceUnit / rdfs:label ?gene_name_label .<br>FILTER (CONTAINS(LCASE(STR(<br>?gene_name_label)), LCASE("apoc1"))) }
Q7	PREFIX up:<http://purl.uniprot.org/core/><br>PREFIX genex:<http://purl.org/genex#><br>PREFIX obo:<http://purl.obolibrary.org/obo/><br>PREFIX orth: <http://purl.org/net/orth#><br>select (count(?anatEntity) as ?c) ?specName ?confidence {<br>?seq a orth:Gene.<br>?expr genex:hasSequenceUnit ?seq.<br>?expr genex:hasConfidenceLevel ?confidence.<br>?seq rdfs:label ?geneName .<br>?expr genex:hasExpressionCondition ?cond.<br>?cond genex:hasAnatomicalEntity ?anatEntity.<br>?cond obo:RO_0002162 ?taxon. #in taxon property.<br>?taxon up:commonName ?specName.<br>FILTER (LCASE(?geneName) = 'apoc1')}<br>group by ?taxon ?confidence<br>order by ?specName	PREFIX genex: <http://purl.org/genex#><br>PREFIX rdfs: <http://www.w3.org/2000/01/rdf-schema#><br>PREFIX orth: <http://purl.org/net/orth#><br>PREFIX obo: <http://purl.obolibrary.org/obo/><br>PREFIX core: <http://purl.uniprot.org/core/><br><br>SELECT ?taxonomy_common_name (COUNT(?anatomical_entity_name) AS ?number_of_anatomical_entities)<br>(MIN(?expression_fdr_pvalue) AS ?min_pvalue)<br>WHERE { ?Expression a genex:Expression ;<br>genex:hasExpressionCondition / genex:hasAnatomicalEntity / rdfs:label ?anatomical_entity_name ;<br>genex:hasSequenceUnit / orth:organism / obo:RO_0002162 / core:commonName ?taxonomy_common_name ;<br>genex:hasFDRpvalue ?expression_fdr_pvalue ;<br>genex:hasSequenceUnit / rdfs:label ?gene_name .<br>FILTER(CONTAINS(LCASE(STR(<br>?gene_name)), LCASE("apoc1"))) }<br>GROUP BY ?taxonomy_common_name<br>ORDER BY ?taxonomy_common_name
Q8	PREFIX up:<http://purl.uniprot.org/core/><br>PREFIX genex:<http://purl.org/genex#><br>PREFIX obo:<http://purl.obolibrary.org/obo/><br>PREFIX orth: <http://purl.org/net/orth#><br>select distinct ?anatEntity ?anatName {<br>?seq a orth:Gene.<br>?expr genex:hasSequenceUnit ?seq.<br>?seq rdfs:label ?geneName .<br>?expr genex:hasExpressionCondition ?cond.<br>?cond genex:hasAnatomicalEntity ?anatEntity.<br>?anatEntity rdfs:label ?anatName.<br>?cond obo:RO_0002162 ?taxon. #in taxon property.<br>?taxon up:commonName 'rat'.<br>FILTER (LCASE(?geneName) = 'apoc1')}	PREFIX genex: <http://purl.org/genex#><br>PREFIX rdfs: <http://www.w3.org/2000/01/rdf-schema#><br>PREFIX orth: <http://purl.org/net/orth#><br>PREFIX obo: <http://purl.obolibrary.org/obo/><br>PREFIX core: <http://purl.uniprot.org/core/><br><br>SELECT DISTINCT ?anatomical_entity_name<br>WHERE { ?Expression a genex:Expression ;<br>genex:hasExpressionCondition / genex:hasAnatomicalEntity / rdfs:label ?anatomical_entity_name ;<br>genex:hasSequenceUnit / rdfs:label ?gene_name ;<br>genex:hasSequenceUnit / orth:organism / obo:RO_0002162 / core:commonName ?taxonomy_common_name .<br>FILTER (CONTAINS(LCASE(STR(<br>?gene_name)), LCASE("apoc1")))<br>FILTER (CONTAINS(LCASE(STR(<br>?taxonomy_common_name)), LCASE("rat"))) }

Table S.10:

	Ground truth	Our Method
Q9	PREFIX up:<http://purl.uniprot.org/core/><br>PREFIX genex:<http://purl.org/genex#><br>PREFIX obo:<http://purl.obolibrary.org/obo/><br>PREFIX rdfs: <http://www.w3.org/2000/01/rdf-schema#><br>PREFIX orth: <http://purl.org/net/orth#><br>select ?anatEntity ?anatName {<br>?anatEntity a genex:AnatomicalEntity.<br>?anatEntity rdfs:label ?anatName.<br>FILTER<br>OT EXISTS {<br>?seq a orth:Gene.<br>?expr genex:hasSequenceUnit ?seq.<br>?seq rdfs:label ?geneName .<br>?expr genex:hasExpressionCondition ?cond.<br>?cond genex:hasAnatomicalEntity ?anatEntity.<br>?anatEntity a genex:AnatomicalEntity.<br>FILTER (LCASE(?geneName) = 'apoc1') }<br>}	PREFIX genex: <http://purl.org/genex#><br>PREFIX rdfs: <http://www.w3.org/2000/01/rdf-schema#><br>PREFIX orth: <http://purl.org/net/orth#><br><br>SELECT DISTINCT ?anatomical_entity_name<br>WHERE {<br>?Expression a genex:Expression ;<br>genex:hasExpressionCondition / genex:hasAnatomicalEntity / rdfs:label ?anatomical_entity_name .<br>OPTIONAL { ?Expression genex:hasSequenceUnit / rdfs:label ?gene_name .<br>FILTER (CONTAINS(LCASE(STR(?gene_name)), LCASE("apoc1"))) }<br>FILTER(!BOUND(?gene_name)) }
Q10	PREFIX up:<http://purl.uniprot.org/core/><br>PREFIX genex:<http://purl.org/genex#><br>PREFIX obo:<http://purl.obolibrary.org/obo/><br>PREFIX orth: <http://purl.org/net/orth#><br>select distinct ?anatEntity ?anatName {<br>?seq a orth:Gene.<br>?expr genex:hasSequenceUnit ?seq.<br>?seq rdfs:label ?geneName .<br>?expr genex:hasExpressionCondition ?cond.<br>?cond genex:hasAnatomicalEntity ?anatEntity.<br>?anatEntity rdfs:label ?anatName.<br>?cond genex:hasDevelopmentalStage ?stage.<br>?stage rdfs:label ?stageName.<br>?cond obo:RO_0002162 ?taxon. #in taxon property.<br>?taxon up:commonName 'mouse'.<br>FILTER (contains(?stageName,"adult") && LCASE(?geneName) = 'apoc1')}	PREFIX genex: <http://purl.org/genex#><br>PREFIX rdfs: <http://www.w3.org/2000/01/rdf-schema#><br>PREFIX efo: <http://www.ebi.ac.uk/efo/><br>PREFIX orth: <http://purl.org/net/orth#><br><br>SELECT DISTINCT ?anatomical_entity_name<br>WHERE {<br>?Expression a genex:Expression ;<br>genex:hasExpressionCondition / genex:hasAnatomicalEntity / rdfs:label ?anatomical_entity_name ;<br>genex:hasExpressionCondition / genex:hasDevelopmentalStage / rdfs:label ?developmental_stage;<br>genex:hasSequenceUnit / rdfs:label ?gene_name .<br>FILTER (CONTAINS(LCASE(STR(<br>?developmental_stage)), LCASE("adult stage")))<br>FILTER (CONTAINS(LCASE(STR(<br>?gene_name)), LCASE("apoc1"))) }

Table S.11:

Bibliography

[1] Eric Jain, Amos Bairoch, Severine Duvaud, Isabelle Phan, Nicole Redaschi, Baris E. Suzek, Maria J. Martin, Peter McGarvey, and Elisabeth Gasteiger. Infrastructure for the life sciences: design and implementation of the UniProt website. *BMC Bioinformatics*, 10(1):136, May 2009.

[2] Parit Bansal, Anne Morgat, Kristian B Axelsen, Venkatesh Muthukrishnan, Elisabeth Coudert, Lucila Aimo, Nevila Hyka-Nouspikel, Elisabeth Gasteiger, Arnaud Kerhornou, Teresa Batista Neto, Monica Pozzato, Marie-Claude Blatter, Alex Ignatchenko, Nicole Redaschi, and Alan Bridge. Rhea, the reaction knowledgebase in 2022. *Nucleic Acids Research*, 50(D1):D693–D700, 11 2021.

[3] Frederic B Bastian, Julien Roux, Anne Niknejad, Aurélie Comte, Sara S Fonseca Costa, Tarcisio Mendes de Farias, Sébastien Moretti, Gilles Parmentier, Valentine Rech de Laval, Marta Rosikiewicz, Julien Wollbrett, Amina Echchiki, Angélique Escoriza, Walid H Gharib, Mar Gonzales-Porta, Yohan Jarosz, Balazs Laurenczy, Philippe Moret, Emilie Person, Patrick Roelli, Komal Sanjeev, Mathieu Seppey, and Marc Robinson-Rechavi. The Bgee suite: integrated curated expression atlas and comparative transcriptomics in animals. *Nucleic Acids Research*, 49(D1):D831–D847, 10 2020.

[4] Shuichi Kawashima, Toshiaki Katayama, Hideki Hatanaka, Tatsuya Kushida, and Toshihisa Takagi. NBDC RDF portal: a comprehensive repository for semantic data in life sciences. *Database*, 2018:bay123, 12 2018.

[5] Toshiaki Katayama and et al. RDF-config. `https://github.com/dbcls/rdf-config`. Accessed: 2025-06-26.
